# Monitoring of pyrethroid resistance in *Aedes aegypti*: first report of double and triple *kdr* mutations in Buenos Aires Province

**DOI:** 10.1186/s13071-024-06547-4

**Published:** 2024-11-09

**Authors:** Alberto N. Barrera-Illanes, Lorena Ledesma, Agustin Alvarez-Costa, Agustín Balsalobre, Corina Juliana Toloza, Agustín Hernandez-Maiztegui, Andrea Jait, Ivana Sierra, María Victoria Micieli, Mariana Manteca-Acosta, Sheila Ons

**Affiliations:** 1 Laboratorio de Neurobiología de Insectos (LNI), Centro Regional de Estudios Genómicos, Facultad de Ciencias Exactas, CENEXA, CONICET, Universidad Nacional de La Plata, La Plata, Buenos Aires Argentina; 2 Centro Nacional de Diagnóstico e Investigación en Endemo-Epidemias, CeNDIE, ANLIS Malbrán, Ministerio de Salud de la Nación, Buenos Aires, Argentina; 3 Laboratorio de Insectos Vectores, Centro de Estudios Parasitológicos y Vectores (CEPAVE CONICET CCT-La Plata-UNLP), La Plata, Buenos Aires Argentina; 4 Dirección de Salud Ambiental, Dirección Provincial de Epidemiología, Ministerio de Salud de la Provincia de Buenos Aires, La Plata, Buenos Aires Argentina

**Keywords:** Dengue, Mosquito, High-resolution melting, Vector management, Insecticide

## Abstract

**Background:**

Dengue is an emerging disease in Argentina due to the colonization of *Aedes aegypti*, the mosquito vector. Buenos Aires Province is the biggest and most populated district in Argentina, suffering dengue outbreaks of growing magnitude. During epidemic periods, pyrethroid insecticides are used in this country to control adult mosquitoes. Pyrethroid resistance in dengue vectors has been reported worldwide, making it necessary to implement resistance management strategies. The voltage-gated sodium channel is the target site of pyrethroids. Mutations in the gene encoding this protein, called *kdr* mutations, are usually the molecular cause of pyrethroid resistance in insects. In *Ae. aegypti* from the Americas, three *kdr* substitutions were described: V410L, V1016I, and F1534C. The diagnostic of *kdr* mutations is recommended for the early detection of pyrethroid resistance as well as the consequent planning of evidence-based control policies.

**Methods:**

We distributed ovitraps across 16 localities in Buenos Aires Province, collecting 22,123 eggs. A total of 522 mosquitoes were genotyped in positions 1016 and 1534 of voltage-gated channel using multiplex high-resolution melting and/or TaqMan probe methods. A subset of 449 samples was also genotyped by a singleplex high-resolution melting method developed ad hoc and/or Sanger sequencing.

**Results:**

We have documented, for the first time to our knowledge in the central region of Argentina, the presence of the 1016Ikdr + 1534Ckdr allele. Additionally, our study reports the first identification of the V410L mutation in central Argentina. These results underscore a growing trend of pyrethroid resistance in *Ae. aegypti*, fueled by the widespread use of these insecticides.

**Conclusions:**

We detected 1016Ikdr + 1534Ckdr and 410Lkdr mutations in central Argentina for the first time and improved the processivity and accuracy of *kdr* genotyping methods. The results are both a tool for resistance monitoring and a sign of alarm to direct efforts towards finding sustainable methods for vector control to complement or replace pyrethroids. Joint efforts between academia and authorities to develop and implement public policies for vector control are a productive way to transfer scientific results for their application in public health.

**Graphical Abstract:**

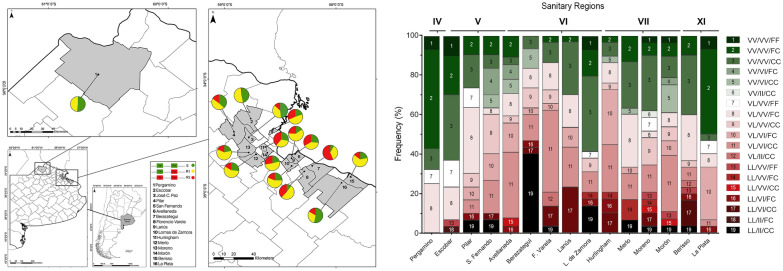

**Supplementary Information:**

The online version contains supplementary material available at 10.1186/s13071-024-06547-4.

## Background

*Aedes aegypti*, a vector of arboviruses such as dengue, Zika, and chikungunya, has re-colonized northeast Argentina since 1987 [1]. The Buenos Aires Province (BAP), the biggest and most populated district in Argentina, was reinfested in 1991 [2], with high levels of mosquito dispersion found in private dwellings. Five dengue outbreaks of increasing magnitude occurred in 2009, 2016, 2020, 2023, and 2024. In the 2023 outbreak, the situation was aggravated by the simultaneous circulation of two dengue virus serotypes (DENV-1 and DENV-2), whereas in 2024 DENV-1, DENV-2, and DENV-3 have been circulating, increasing the risk of severe cases. Nearly 10,000 confirmed dengue cases were reported in the 2022–2023 outbreak in BAP, whereas almost 100,000 confirmed cases have been reported for 2023–2024 until epidemiological week 20–2024 (https://www.gba.gob.ar/saludprovincia/boletines_epidemiologicos/1000?page=45). These numbers illustrate the extraordinary expansion of dengue in the region and are the cause of serious concern for *Ae. aegypti* control.

Vector control is the method of choice for reducing transmission of arbovirus and other vector-borne diseases. Elimination of domestic breeding sites and the use of larvicides are measures that should be implemented throughout the year to minimize arbovirus outbreaks [3]. The use of adulticides is recommended to be restricted to epidemic periods [3]; for this, neurotoxic pyrethroids are the most widely used compounds given their favorable toxicological properties. Even though the organophosphate malathion is used in cases of high pyrethroid resistance in the Americas, its application in Argentina was banned for use in dwellings, leaving pyrethroids as the only insecticide allowed in this country for public health applications [4]. This situation is challenging, given that the continued use of neurotoxics fueled the emergence of resistant vector populations worldwide. For *Ae. aegypti*, pyrethroid resistance was detected in the whole distribution range of the species, making this group of insecticides obsolete in a lot of regions. For mosquito control, the World Health Organization (WHO) recommends integrated management, which must incorporate resistance monitoring and management strategies [3]. As an example of this, a systematic plan, adapted to the territories for insecticide surveillance in mosquitoes, was recently proposed [5]. This plan proposes the stratification of the level of risk in different regions, by evaluating the toxicological profile and molecular markers in local vector populations. This kind of action will allow designing evidence-based public policies of vector control, adjusted to the situation in the different zones of the territory. For this, the availability of high-throughput methods for detecting genetic mutations associated with insecticide resistance will be essential.

Pyrethroid insecticides exert a toxic action through their target site, the voltage-gated sodium channel (VGSC), present in the membrane of excitable cells [6]. Insecticide resistance is a complex trait; one of the main causes of pyrethroid resistance is the existence of point mutations in the *vgsc* gene, called knockdown resistance mutations (*kdr*). These mutations are usually located in regions of the molecule that interact with pyrethroids (pyrethroid sites 1 and 2) [7]. Several *kdr* mutations have been described in *Ae. aegypti* [8]; in the Americas region, the most important ones, given their reported association with pyrethroid resistance, are Val to Ile in position 1016 (V1016I) and Phe to Cys in position 1534 (F1534C) of VGSC protein [9]. More recently, Val to Leu in position 410 (V410L) has been described in Mexico, Colombia, Brazil, and the USA [10–13]. The association of V410L *kdr* mutation with insecticide resistance has not been extensively studied to date, even though recent results showed an increase of the resistance to pyrethroids in the field, with a significant interaction with distance of spraying application [14].

In a recent work [9], we demonstrated, for the first time to our knowledge, the presence of 1534C*kdr* mutation in BAP, while 410L*kdr* or 1016I*kdr* have not been reported in the central region of Argentina to date. Considering 1016 and 1534 positions, four *kdr* alleles are possible: 1016 V + 1534F (sensitive: S); 1016 V + 1534C*kdr* (resistant 1: R1); 1016I*kdr* + 1534C *kdr* (resistant 2: R2); 1016I*kdr* + 1534F (resistant 3: R3). Whereas R1 and R2 alleles are widely extended in Latin America, R3 has been detected with a very low frequency (≤ 0.1% of the samples) in Brazil [10], Mexico [15], and Florida [16] but not in Argentina to date. In our previous study analyzing samples collected in 2018–2019 [9], 1016I*kdr* + 1534C *kdr* allele was detected in northern Argentina but not in BAP. This could be due to the more recent history of dengue in BAP, where the first outbreak was reported in 2016. From then, outbreaks of increasing magnitude occurred in this province in 2020, 2023, and 2024. This scenario allows us to study the evolution of *kdr* genotypes in *Ae. aegypti* after two outbreaks in which pyrethroid-based adulticide campaigns were carried out with permethrin, deltamethrin, and cypermethrin.

The estimation of frequencies of *kdr* mutations in natural populations is relevant to detecting the emergence of resistance at early stages and estimating the level of resistance in a population. This, in conjunction with information on toxicological tests with insecticides, provides information that is necessary for planning public policies and evaluating control interventions [5]. Vector control actions should be adapted to the situation of insecticide resistance in each territory. The use of TaqMan probes has been adopted as the gold standard method for genotyping *kdr* in *Ae. aegypti*. This is a reliable and robust method, even though probes are expensive, and multiplexing is not possible, making it necessary to use a single reaction for each mutation. This makes TaqMan a suboptimal method for implementation in wide regional control campaigns, especially in low- or middle-income countries, which are those more affected by mosquito-transmitted diseases. We recently developed a multiplex high-resolution melting (mHRM) assay for rapid genotyping of both 1016 and 1534 positions in *Ae. aegypti vgsc* gene [9], which can be used to replace TaqMan, considering performance and cost.

Here, we document, for the first time in the central region of Argentina to our knowledge, the presence of the 1016Ikdr + 1534Ckdr allele, probably because of the selective pressure exerted by pyrethroids during recent dengue outbreaks. Additionally, our study reports the first identification of the V410L mutation in central Argentina to our knowledge. These results underscore a growing trend of pyrethroid resistance in *Ae. aegypt*i, fueled by the widespread use of these insecticides. Since pyrethroids are the only insecticides approved for domestic and sanitary use in Argentina, the perspectives of our study have critical implications for evidence-based public health policies of dengue vector control.

## Methods

### Study area

BAP is the biggest and most populated district in Argentina, considering both its surface (307,571 km^2^) and the number of inhabitants (around 17.5 million), representing 38% of the total population of the country and one-third of the gross domestic product. The sanitary system in BAP is divided into 12 Sanitary Regions (SRs), each composed of several localities (Municipalities). Each SR manages and monitors health policies at the local scale. Pyrethroids used by the health system for mosquito control are permethrin and, to a lesser extent, deltamethrin and cypermethrin. The climate is temperate and humid to subhumid, with a southwest-northeast gradient. In western areas, rainfall ranges between 400 and 500 mm annually, while in eastern areas, it exceeds 1000 mm annually, being more abundant from October to March.

### Sample collection

Priorities proposed by BAP health authorities to include particular localities in the study were the registered outbreaks of dengue cases between 2016 and 2023 along with the consequent control of adult mosquitoes through spatial spraying. The samples were collected in 2023 in 16 localities of BAP, belonging to 5 different SRs (Table 1): Avellaneda (34°39′45″S 58°22′04″W); Berisso (34°52′00″S 57°52′00″W); Berazategui (34°46′04″S 58°12′47″W); Escobar (34°21′00″S 58°46′00″W); Florencio Varela (34°49′00″S 58°17′00″W); Hurlingham (34°36′00″S 58°38′00″W); José C. Paz (34°31′S 58°47′W); Lanús (34°42′S 58°24′W); Lomas de Zamora (34°46′00″S 58°24′00″W); La Plata (34°55′00″S 57°57′00″W); Merlo (34°40′00″S 58°43′00″W); Moreno (34°39′00″S 58°47′00″W); Morón (34°39′00″S 58°37′00″W); Pilar (34°28′00″S 58°55′00″W); Pergamino (33°53′00″S 60°35′00″W); San Fernando (34°27′00″S 58°34′00″W).

**Table 1:** Frequencies of genotypes considering the V1016I and F1534C *kdr* mutations in *Aedes aegypti* from Buenos Aires

Sanitary Region	Municipality	Dengue cases	Genotype frequencies (confidence interval)						N	HWE test	
		/10,000 inhabitants 2023 &	SS	SR1	R1R1	SR2	R1R2	R2R2		X^2^	p value
IV	Pergamino	0	0.14 (0.01–0.27)	0.76 (0.61–0.91)	0.1 (0–0.22)	0	0	0	29	11.08	0.001
V	Escobar	3.39	0.27 (0.12–0.42)	0.37 (0.21–0.53)	0.33 (0.17–0.49)	0.03 (0–0.12)	0 (0–0.08)	0 (0–0.08)	30	2.74	0.43
	Jose C. Paz	1.51	0.23 (0.08–0.38)	0.37 (0.21–0.53)	0.13 (0–0.26)	0.13 (0–0.26)	0.13 (0–0.26)	0 (0–0.08)	30	0.91	0.82
	Pilar	1.97	0.10 (0–0.22)	0.43 (0.26–0.60)	0.23 (0.08–0.37)	0.10 (0–0.22)	0.13 (0–0.26)	0 (0–0.08)	30	1.13	0.77
	San Fernando	4.23	0 (0–0.08)	0.13 (0–0.26)	0.30 (0.14–0.46)	0.27 (0.12–0.42)	0.20 (0.06–0.34)	0.10 (0–0.23)	30	11.1	0.01
VI	Avellaneda	6.36	0 (0–0.08)	0.23 (0.08–0.37)	0.10 (0–0.22)	0.30 (0.14–0.46)	0.37 (0.21–0.53)	0 (0–0.08)	30	9.86	0.02
	Berazategui	46.87	0 (0–0.08)	0.10 (0–0.22)	0.17 (0.03–0.31)	0.07 (0–0.18)	0.27 (0.12–0.42)	0.40 (0.23–0.57)	30	3.74	0.29
	Florencio Varela	6.27	0 (0–0.08)	0.17 (0.03–0.31)	0.17 (0.03–0.31)	0.03 (0–0.13)	0.50 (0.33–0.67)	0.10 (0–0.22)	29	3.44	0.22
	Lanús	4.48	0 (0–0.07)	0.21 (0.07–0.35)	0.33 (0.15–0.45)	0.12 (0–0.20)	0.39 (0.23–0.55)	0 (0–0.07)	33	4.54	0.21
	Lomas de Zamora	3.25	0.12 (0–0.24)	0.24 (0.10–0.38)	0.38 (0.22–0.54)	0.03 (0–0.12)	0.12 (0–0.24)	0.12 (0–0.24)	34	11.66	0.008
VII	Hurlingham	3.1	0 (0–0.07)	0.17 (0.05–0.29)	0.06 (0–0.15)	0.39 (0.24–0.54)	0.39 (0.24–0.54)	0 (0–0.07)	36	16.51	0.001
	Merlo	6.3	0 (0–0.08)	0.50 (0.33–0.67)	0.23 (0.08–0.38)	0.03 (0–0.12)	0.17 (0.03–0.31)	0.07 (0–0.18)	30	9.27	0.03
	Moreno	3.6	0.13 (0–0.26)	0.13 (0–0.26)	0.43 (0.26–0.60)	0.03 (0–0.12)	0.20 (0.06–0.34)	0.07 (0–0.18)	30	8.78	0.03
	Morón	8.77	0.06 (0–0.16)	0.19 (0.05–0.33)	0.23 (0.09–0.37)	0.10 (0–0.22)	0.39 (0.25–0.55)	0.03 (0–0.12)	31	2.63	0.45
XI	Berisso	0	0.03 (0–0.12)	0.33 (0.17–0.49)	0.37 (0.21–0.53)	0.03 (0–0.12)	0.20 (0.06–0.34)	0.03 (0–0.12)	30	1.21	0.75
	La Plata	0.61	0.13 (0–0.26)	0.50 (0.33–0.67)	0.03 (0–0.12)	0.30 (0.14–0.46)	0.03 (0–0.12)	0 (0–0.08)	30	11.16	0.01

^a^Considering epidemiological weeks 1–18

*Aedes aegypti* eggs were collected using 158 ovitraps during the peak oviposition activity period (February and March) in 2023; a total of 22,123 eggs were collected. Ovitraps were deposited outdoors in 79 primary health care centers and hospitals (between 3 and 8 ovitraps per site) by trained agents of BAP Ministry of Health. The ovitraps consisted of 500-ml black plastic containers filled with 200 ml water, each equipped with five oviposition substrates. The substrates were replaced weekly for 6 weeks. The collected eggs were transported to either National Center for Diagnosis and Research in Endemo-epidemics (CENDIE-ANLIS-Malbrán) or the Center for Parasitological and Vector Studies (CEPAVE-UNLP-CONICET). Eggs collected per locality were counted under a stereoscopic microscope. Under controlled laboratory conditions (temperature: 26º ± 2  C, humidity: 70 ± 5%, and photoperiod: 12:12 light: dark), oviposition substrates sourced from each location were immersed in plastic trays filled with dechlorinated water and yeast. Upon egg hatching, yeast was provided to the larvae until reaching the pupal stage. Subsequently, the pupae were transferred to plastic water containers. From the total number of eggs that reached the adult stage, between 29 and 35 individuals/locality were randomly selected, individually isolated, and preserved at − 80 °C in absolute ethanol until DNA extraction.

The mosquito heads were removed, and the remaining body parts were transferred to 1.5-ml tubes containing 100 µl of pre-warmed grinding buffer (5 M NaCl, 0.5 M EDTA, 1 M Tris base pH 7.5, 10% SDS). Samples were homogenized with sterile pistils, and 3 μl Proteinase K (20 μg/ml) was added, followed by incubation at 55 °C for 15 min. Subsequently, 14 μl 8 M potassium acetate was added to each tube and mixed thoroughly. The mixture was placed on ice for 15 min before centrifugation at 13,000 rpm for 15 min. DNA from the supernatant was precipitated with absolute ethanol. The DNA concentration and quality extracted from each individual were estimated with a spectrophotometer and diluted until reaching a final concentration of 20 ng/μl. Samples with a 260:230 ratio > 1.5 and 260:280 ratio between 1.8 and 2.2 were used for genotyping.

### mHRM, HRM, TaqMan, and Sanger assays for genotyping 410, 1016, and 1534 positions

For genotyping 1016 and 1534 positions, mHRM assays were performed as described previously [9]. Briefly, 2 µl of individual genomic DNA was used for amplification in a final 12 μl reaction volume that included 6 μl Brilliant HRM Ultra-fast Loci Master Mix (Applied Biosystems) and 200 nM of each primer (see primer sequences in Table 2). The qPCR protocol comprised a 3-min denaturation step at 95 °C, followed by 40 cycles of 5 s denaturation at 95 °C and a 30-s annealing extension at 62 °C. Subsequently, the melting curves obtained were analyzed using AriaMX 1.5 software (Applied Biosystems). Results were determined with GENOMOS application (see below) and visualization of melting curves. For genotyping 410 position, the reaction mix had a final reaction volume of 10 μl, including 5 μl of Brilliant HRM Ultra-fast Loci Master Mix (Applied Biosystems) and 200 nM of each primer (see primer sequences in Table 2). The qPCR protocol was identical to the one described above. Melting curves were visualized with AriaMX 1.5 software (Applied Biosystems).

**Table 2:** Primer sequences

	Primer/probe name	Sequence	Use	
F1534C	Ae1534qPCRFwGC2	CGGCGGCGGTGTACCTCTACTTTGTGTTCTTCA	mHRM	
	Ae1534qPCRRvGC	CGGGTCGTTCTTCACGCTGCCGCCGCCGG	mHRM	
	AHUADFA_F	CGAGACCAACATCTACATGTACCT	TaqMan	
	AHUADFA_R	GATGATGACACCGATGAACAGATTC	TaqMan	
	AHUADFA_V	VIC-AACGACCCGCAGATGA-NFQ	TaqMan	
	AHUADFA_M	FAM-ACGACCCGAAGATGA-NFQ	TaqMan	
V1016I	Ae1016G2_Fw	CAAATTGTTTCCCACTCGCACAG	mHRM	
	Ae1016GqPCRRev	AGTAAGTATTCCGTTTGGAAGTTC	mHRM	
	AHS1DL6_F	CGTGCTAACCGACAAATTGTTTCC	TaqMan	
	AHS1DL6_R	GACAAAAGCAAGGCTAAGAAAAGGT	TaqMan	
	AHS1DL6_V	FAM-CCCGCACAGGTACTTA-NFQ	TaqMan	
	AHS1DL6_M	VIC-CCGCACAGATACTTA-NFQ	TaqMan	
V410L	Ae410Fw	GTGTTACGATCAGCTGGACC	Sanger sequencing	
	Ae410Rv	CCCGAAGCGCTTCTTCCTCG	Sanger sequencing	
	Ae410Fw2	TGTGATTATCTTCTTGGGTTCGT	HRM	
	Ae410Rv2	GCGACAATGGCCAAGATCAA	HRM	

A subset of samples was genotyped with both mHRM and TaqMan to evaluate whether mHRM has a similar performance to the gold standard method (see Results). For TaqMan-based genotyping, 5 μl Master Mix qPCR probe (Productos Bio-lógicos), 2 μl DNA template (20 ng/μl), and 0.25 μl of a mix of primers and probe (Thermo Fisher) were used in each reaction. Custom TaqMan SNP Genotyping Assays (Thermo Fisher, AHS1DL6 for V1016I or AHUADFA for F1534C) were used. The reaction started with an activation at 95 °C for 5 min, followed by 40 cycles of 95 °C for 15 s and 60 °C for 90 s with a signal acquisition in both the FAM and HEX channels at the end of the annealing/extension step. For mHRM, HRM, and TaqMan assays, samples of known genotypes were included in each plate in triplicate as standards for comparisons with unknown samples.

For Sanger sequencing, genomic DNAs were used as the template for PCR reactions with primers Ae410Fw and Ae410Rv (Table 2), as well as the polymerase GoTaq (Promega), according to manufacturer’s instructions. The cycling conditions included an initial denaturation at 95 °C for 5 min, followed by 40 cycles of 30 s at 95 °C (denaturation) and 30 s at 60 °C, concluding with a final extension step at 72 °C for 5 min. Samples were sequenced at Macrogen (Seoul, Korea) using the Ae410Rv primer.

### GENOMOS application development

We developed a desktop application (GENOMOS) to improve the processivity of the mHRM result analysis and their interpretation, with a user-friendly graphical interface, which can be downloaded from https://github.com/gonzalohdominguez/GENOMOS. It has been developed in the Python programming language, designed with Qt Creator using the PySide2 module. GENOMOS application determines the absolute difference between melting temperature (MT) of the three standards (two homozygous and heterozygous genotypes) for 1016 and 1534 positions and the MT of each sample. The lower difference will be determined as the probable genotype of the unknown sample. The application provides the results in percentages of allele and genotype distribution and automatically generates pie charts for better visualization.

### Statistical analyses

The frequency of each mutation was calculated as the sum of two times the number of homozygotes and the number of heterozygotes carrying that mutation divided by two times the sample size. The confidence interval (95%) was calculated as the Wald interval [17]. Goodness of fit to Hardy-Weinberg equilibrium (HWE) was calculated by Chi-square test with 1 (for Pergamino) or 3 (for the rest of the localities) degrees of freedom. Pearson’s correlation coefficient was calculated with GraphPad software.

## Results

**Fig. 1:**
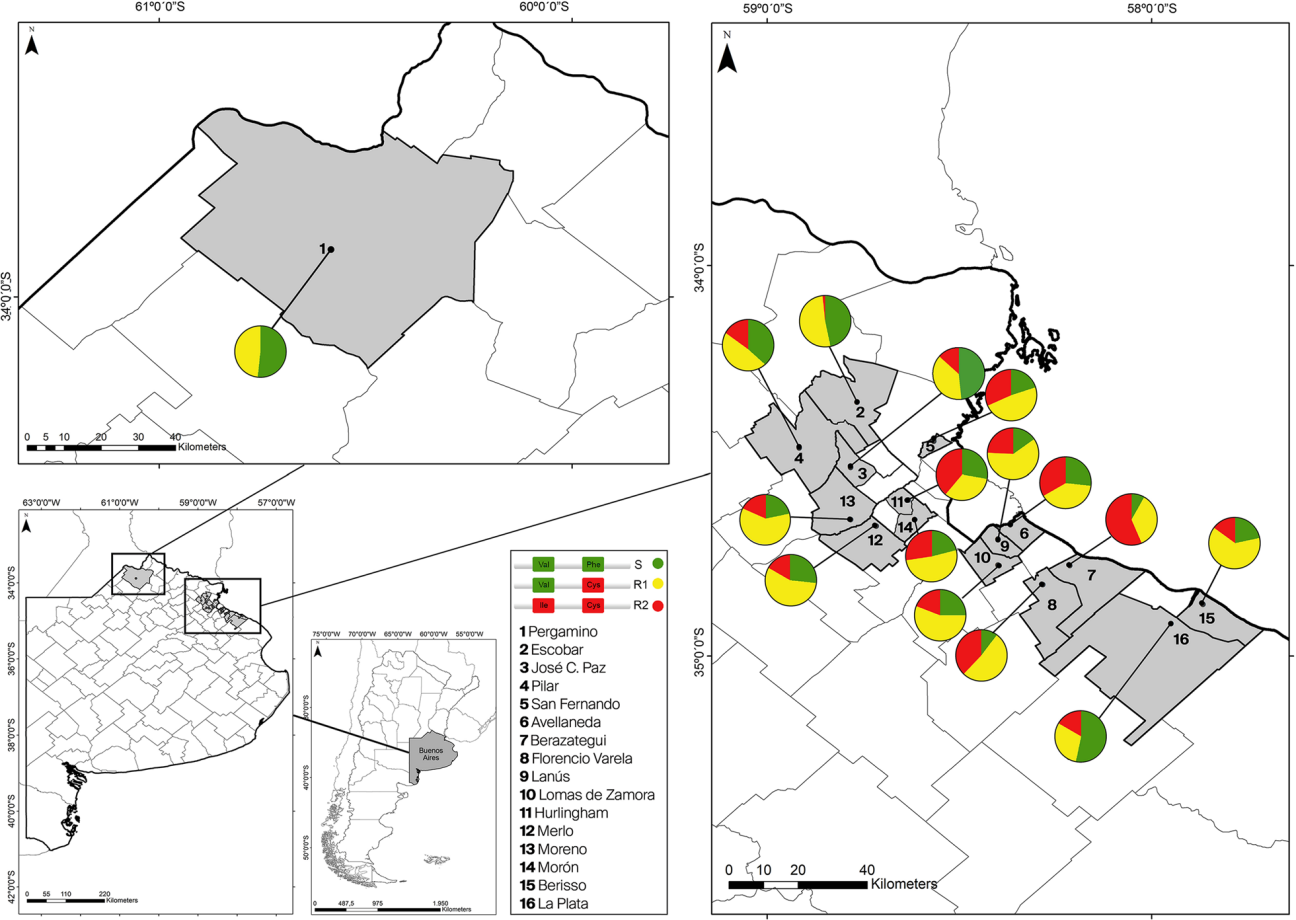
Distribution of the knockdown resistance (*kdr*) alleles considering positions 1016 and 1534 in *Aedes aegypti* from localities belonging to Buenos Aires Province

A total of 522 mosquitoes randomly selected from 16 localities belonging to 5 SRs were genotyped for 1016 and 1534 positions (Table 1; Fig. 1) using mHRM and/or TaqMan probes. The genotypes of 274 samples were determined by using both techniques to compare the performance of mHRM and TaqMan for genotyping 1016 and 1534 positions. We found a 100% agreement between both methods.

All the populations under study presented 1016 V + 1534F and 1016 V + 1534Ckdr alleles (Fig. 1). We detected 1016Ikdr + 1534Ckdr in all the municipalities analyzed, except for Pergamino (Table 1, Fig. 1). Globally, the most abundant allele was 1016 V + 1534Ckdr (48.27%) followed by 1016 V + 1534F (28.66%) and finally 1016Ikdr + 1534Ckdr (23.07%). The frequency of the sensitive allele 1016 V + 1534F varied significantly between localities, being the lowest in Berazategui (SR VI) with 8.3% and the highest in La Plata (SR: XI) with 53.3%. The 1016 V + 1534Ckdr allele showed the greatest diversity in frequency, where Berisso (SR: XI) presented the highest value with 63.3%, while La Plata (SR: XI) had the lowest frequency with 30%. For 1016Ikdr + 1534Ckdr, Berazategui (SR: VI) was the locality with the highest frequency (56.7%), while it was not detected in Pergamino (SR: IV) (Table 1).

Genotype frequencies were determined, and the HWE hypothesis was tested in each population under study (Table 1). In every SR there was at least one locality where the assumption of HWE was rejected (Table 1) (SR IV: Pergamino *p* = 0.001; SR V: San Fernando *p* = 0.01; SR VI: Avellaneda *p* = 0.02, Lomas de Zamora *p* = 0.008; SR VII: Hurlingham *p* = 0.001, Merlo *p* = 0.03, Moreno *p* = 0.03; SR XI: La Plata *p* = 0.01). In most of these localities, except for Lomas de Zamora and Moreno, a deficit of homozygous genotypes was the main cause of disequilibrium (Table 1).

**Fig. 2:**
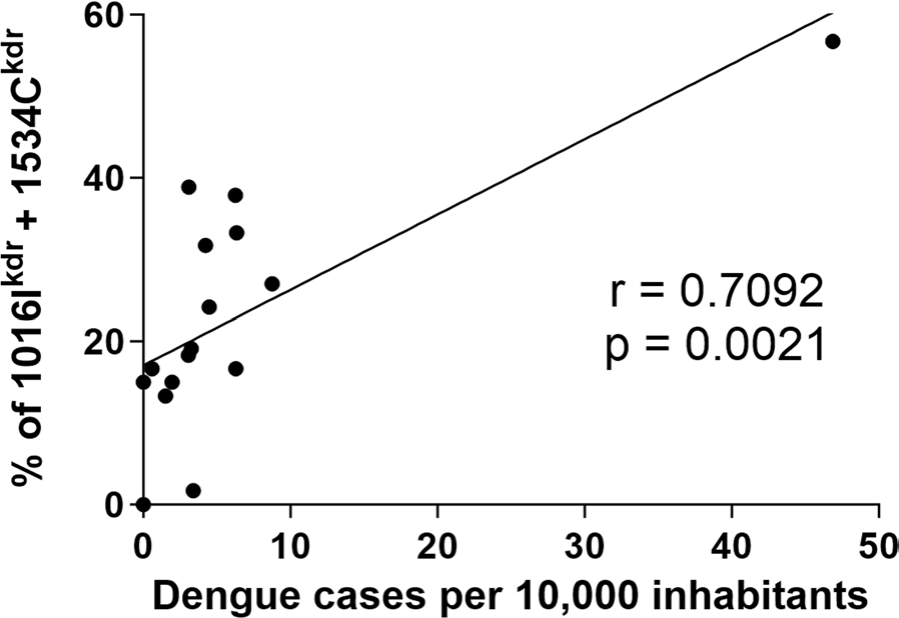
Correlation between dengue cases per 10,000 inhabitants recorded during the 2023 outbreak and percentage of double mutant allele detected in each BAP locality

It was proposed that 1016Ikdr + 1534Ckdr allele emerges in *Ae. aegypti* populations in the presence of 1016 V + 1534Ckdr genetic background in response to pyrethroid selection pressure [18, 19]. In agreement, our present results indicate a recent emerging and rapid expansion of this double mutation in BAP, given that it was not detected in samples collected 4 to 5 years before [9]. We hypothesized that 1016Ikdr + 1534Ckdr allele emerged in BAP in a 1016 V + 1534Ckdr genetic background, which would result in a higher frequency of double mutant allele in those regions under a more intense use of pyrethroids, also correlating with the number of dengue cases per inhabitant. To test this, Pearson’s correlation coefficient and its significance were calculated between dengue cases per inhabitant and 1016Ikdr + 1534Ckdr frequencies for all the localities under study. We observed a positive and significant correlation (*r *= 0.71; *p* < 0.005) (Fig. 2), which is mainly explained by the case of Berazategui, where both dengue cases and double mutation frequency are much higher than for the other localities.

**Fig. 3:**
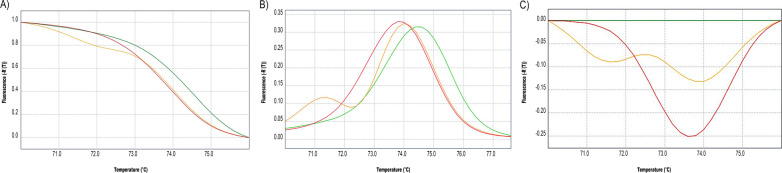
Detection of *kdr* single nucleotide polymorphism in position 410 in the voltage-gated sodium channel gene of *Aedes aegypti* with high-resolution melting (HRM) by melt curve analysis. Alleles are distinguished by changes in the melting temperature (MT) and shape of the melting curves. **A** Normalized **B** Raq derivative and **C** Difference plots. Red: 410L*kdr* homozygous genotype; orange: heterozygous genotype; green: V410

V410L *kdr* mutation in *Ae. aegypti* has been reported in the Americas in recent years [20]. Given the existence of the double mutant allele in BAP reported here, we tested the presence of V410L; for this, we developed a HRM assay, whose representative results are shown in Fig. 3. As expected for the substitution of guanine (in wild-type V410) by thymidine (in 410L*kdr* variant), the MT of the amplicons showed a higher value for the 410VV genotype (74.24 °C ± 0.001 °C; *N* = 140) and a shift of 0.57 °C for the genotype 410 L*kdr*L*kdr* (73.67 °C ± 0.008 °C; *N* = 33). Interestingly, although the heterozygous 410 VL*kdr* samples had a smaller shift of temperature (73.86 °C ± 0.001 °C; *N* = 153), they also presented a different shape in their melting curves compared to both homozygotes (Fig. 3). Altogether, the shifts in temperature and shapes of the melting curves allowed a simple genotype determination for 410 position with HRM.

Four hundred forty-nine samples were genotyped for 410 position (357 by HRM and 92 by Sanger sequencing). The *kdr* substitution V410L was detected at different frequencies in all the localities under study (Table 3). The three possible variants (VV410, VL410, and LL410) were found in all the localities analyzed, except for Pergamino, where the 410Lkdr mutation was not detected in homozygosis (Table 3). This mutation was found in lower frequency regarding the wild-type V410 in all the localities, except for Berazategui and Florencio Varela (both in SR VI), where 410Lkdr was the more abundant variant (Table 3). All the populations were found to be in a HWE, except Florencio Varela and Lomas de Zamora, both in SR VI.

**Table 3:** Frequencies of genotypes considering the V410L kdr mutation in *Aedes aegypti* from Buenos Aires

Sanitary Region	Municipality	Genotype frequencies (confidence interval)			N	HWE test	
		VV	VL	LL		*X*^2^	*p* value
IV	Pergamino	0.68 (0.52–0.84)	0.32 (0.16–0.48)	0 (0–0.22)	29	0.94	0.33
V	Escobar	0.63 (0.46–0.80)	0.3 (0.14–0.46)	0.07 (0–0.17)	30	0.40	0.52
	Pilar	0.27 (0.11–0.43)	0.63 (0.46–0.80)	0.1 (0–0.17)	30	2.79	0.09
	San Fernando	0.37 (0.2–0.54)	0.52 (0.33–0.67)	0.11 (0–0.23)	27	0.34	0.46
VI	Avellaneda	0.3 (0.14–0.46)	0.63 (0.46–0.80)	0.07 (0–0.18)	29	2.93	0.087
	Berazategui	0.17 (0.03–0.31)	0.37 (0.2–0.54)	0.47 (0.3–0.64)	30	1.13	0.29
	Florencio Varela	0.14 (0.01–0.27)	0.72 (0.58–0.88)	0.14 (0.01–0.27)	28	5,14	0.023
	Lanús	0.3 (0.14–0.46)	0.47 (0.3–0.64)	0.23 (0.08–0.38)	30	1.15	0.28
	Lomas de Zamora	0.59 (0.42–0.76)	0.21 (0.06–0.35)	0.21 (0.06–0.35)	29	7.75	0.005
VII	Hurlingham	0.2 (0.07–0.33)	0.65 (0.49–0.81)	0.14 (0–0.29)	29	2.9	0.09
	Merlo	0.4 (0.23–0.57)	0.43 (0.26–0.6)	0.17 (0.03–0.3)	30	0.21	0.64
	Moreno	0.39 (0.22–0.56)	0.5 (0.33–0.67)	0.11 (0–0,23)	30	0.29	0.58
	Morón	0.42 (0.25–0.59)	0.38 (0.21–0.55)	0.2 (0.06–0.35)	30	1.51	0.22
XI	Berisso	0.4 (0.23–0.57)	0.37 (0.2–0.54)	0.23 (0.08–0.38)	30	1.94	0.16
	La Plata	0.53 (0.36–0.7)	0.43 (0.26–0.6)	0.03 (0–0.13)	28	0.57	0.45

**Fig. 4:**
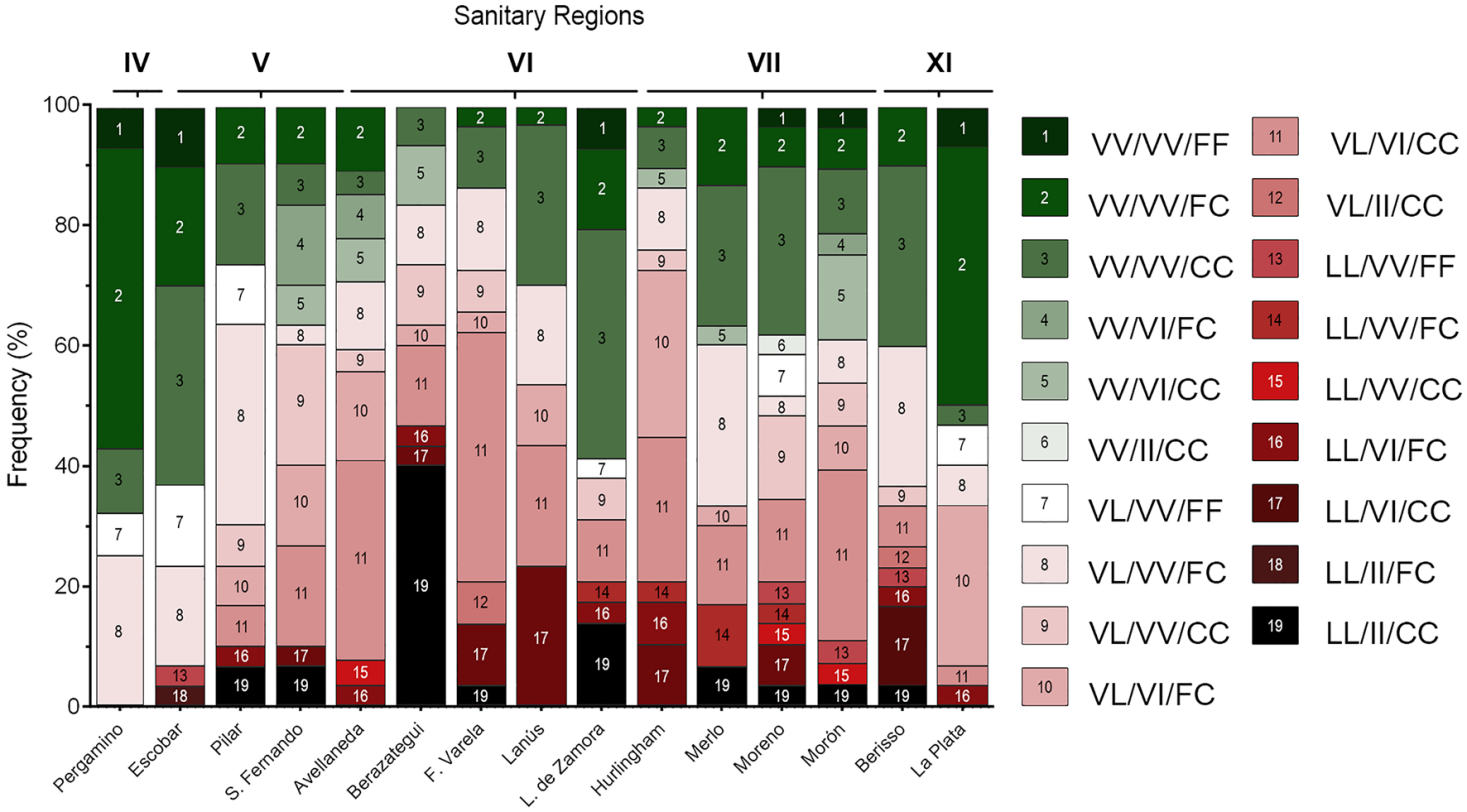
Frequencies of tri-locus (410/1016/1534) genotypes in *Aedes aegypti* populations from Buenos Aires Province

When considering the 3 substitutions under analysis, a total of 19 genotypes were found (Fig. 4 and Supplementary information). Among the localities under study, Morón had the highest variety of genotypes (13), and Pergamino had the lowest (5). The remaining localities presented 7 (Escobar), 8 (La Plata), 9 (Pilar, Berazategui, Florencio Varela, and Lomas de Zamora), 10 (San Fernando, Avellaneda, Hurlingham, and Berisso), and 12 (Moreno) different genotypes (Fig. 4).

The triple *kdr* genotype (LL/II/CC) was detected at low frequencies (≤ 6.7%) in Pilar, San Fernando, Florencio Varela, Merlo, Moreno, Morón, and Berisso and at higher frequencies in two regions from SR VI: Berazategui (40%) and Lomas de Zamora (13.8%). Besides, the triple wild-type (VV/VV/FF) was detected at low frequencies (≤ 10%) in Pergamino, Escobar, Lomas de Zamora, Morón, Moreno, and La Plata but not in the other localities analyzed. Considering all the samples studied, LL/II/CC was present in 5.84%, and VV/VV/FF was detected in 2.5% (Fig. 4; Supplementary information). The more abundant genotype detected was VV/VV/CC (R1R1) in 17% of the samples from BAP. This genotype was followed in abundance by VL/VI/CC (R1R2; 15.4%), VV/VV/FC (R1S, 13.8%) VL/VV/FC (SR1; 13.7%), VL/VI/FC (SR2; 7.75%), and VL/VV/CC (R1R1; 5.45%). The remaining genotypes were detected at frequencies < 5% (Fig. 4 and Supplementary information).

## Discussion

In the current context of increasing dengue outbreaks in South America, it becomes imperative to develop integrated intervention strategies at the regional level. In response to this, RAReP was formed in Argentina in 2021, comprising institutions from the National Ministry of Health and scientific-technical entities. RAReP's mission is to contribute to the monitoring and management of pesticide resistance in arthropod vectors of pathogens by the generation of scientific evidence to optimize efficiency in vector control. Within this framework, here we present the findings of our collaborative multicenter study, which involved both scientific research and governmental teams. Our study aimed to provide essential information on the detection of insecticide resistance, facilitating the planning and evaluation of initiatives to control dengue vector populations. We formed a multicenter working group to conduct an analysis of *kdr* mutations in *Ae. aegypti* from Buenos Aires Province, the most populous district of Argentina. We document, for the first time to our knowledge, the presence of the 1016I*kdr* in the central region of the country. While 410Lkdr has been recently found in four localities in northern Argentina [21], we report for the first time to our knowledge that this substitution is widely extended in the heavily populated Buenos Aires Province in the central region of the country, which led to a total of 19 genotypes being detected; from these, the more abundant was VV/VV/CC, followed by VL/VI/CC. Interestingly, this pattern of abundance was also observed in surveys from the Caribbean region in Colombia [13] and Amazonian [22] and other regions of Brazil [10] with different records of dengue outbreaks. This pattern could indicate a common characteristic of South American *Ae. aegypti* populations where dengue disease is an important health issue, which could be confirmed by further studies.

We observed a positive and significant correlation between dengue cases (a strong indicator of the use of pyrethroids) and the percentage of the double mutant 1016I*kdr* + 1534Ckdr, even though, at the regional level, the more abundant still was 1016 V + 1534Ckdr. Previous reports suggested a sequential selection of *kdr* mutations in *Ae. aegypti*, with an initial emergence of 1534Ckdr in response to DDT/pyrethroids, providing a background for the emergence of the 1016I*kdr* + 1534C*kdr* double mutation in a context of a growing pressure exerted by pyrethroids [19]. Our results reinforce the hypothesis of a sequential selection: given that the 1534C*kdr* but not the double mutant was detected in samples from 2018–2019 [9], we hypothesized that the double mutant emerged recently in BAP and was spreading in response to the sustained use of pyrethroids during the recent dengue outbreaks. It is still possible that the double mutants would have been already present in lower frequencies in 2018–2019 but were not detected in the previous study because of the sample size. Also, double mutant mosquitoes from northern regions could have been introduced in BAP by human-mediated passive transportation and selected by the pressure exerted by pyrethroids, which were used by both health authorities and citizens in private dwellings.

The triple mutant 410Lkdr + 1016Ikdr + 1534Ckdr has a clear fitness disadvantage compared to the single mutant 1534Ckdr in the absence of pyrethroids. Contrarily, in the presence of a pyrethroid such as deltamethrin, the fitness advantage of the triple mutant is enormous [23]. This opposite relationship between the level of protection during periods of insecticide pressure and the fitness cost in the absence of insecticides predicts that the more fumigated localities (those with more dengue cases) will develop higher proportions of double and triple *kdr* individuals. This prediction is confirmed in our work, where the Berazategui locality, with by far the highest number of dengue cases in 2023, was also the locality with a higher abundance of the double and triple mutant alleles.

Interestingly, the genotype frequencies for 1016 and 1534 positions of VGSC do not follow the assumptions of HWE for 50% of the populations studied, indicating that they are under selection pressure. Our results seem to corroborate that 1016Ikdr mutation will emerge in populations with intermediate to high levels of 1534C*kdr* in a period of 5 to 10 years if selection pressure is constant. This was attributed to the rapid emergence of mutations in 1016 and/or 410 positions in a 1534Ckdr genetic background [9, 18].

In a recent work [9], we showed a 100% agreement between Sanger sequencing and mHRM for position 1016 of *Ae. aegypti* VGSC, even though for position 1534 the accuracy of mHRM compared with Sanger was reduced to 85.7%. Here, we showed an improvement in mHRM, reaching 100% agreement with TaqMan probes for both positions, refining the purity of genomic DNA obtained from each mosquito; for this, we used an alternative method and removed the heads of the insects before DNA purification. Also, we developed a simple application with a visual interface (GENOMOS), available for the rapid estimation and visualization of genotypic results for both 1016 and 1534 positions. For 410 position, we developed a singleplex HRM that allows the distinction between genotypes by both MT and the shape of the melting curve. These improvements make mHRM and HRM suitable tools for the timely management of the huge number of samples employed in country-wide determinations of genotype frequencies, during and after dengue outbreaks and insecticidal interventions. The simultaneous detection of three or more positions could be possible by using a multiplex HRM technique [24]. Even though this three-site multiplexing could require a careful setting of the assays, it could be a future improvement for the processivity of *kdr* genotyping.

At the end of the austral summer season in 2024 (March 2024), Argentina, Brazil, and Paraguay concentrated 92% of the cases and 87% of the deaths caused by the dengue virus worldwide, according to the Pan American Health Organization (PAHO; https://www.paho.org/es/noticias/28-3-2024-ops-llama-accion-colectiva-ante-aumento-record-casos-dengue-americas). Particularly, arbovirus transmission in Argentina is a growing concern, given that *Ae. aegypti* populations are expanding across the central regions of the country, being reported in southern localities over time. The finding of new *kdr* mutations in the most densely populated province demands the inclusion of monitoring and management of insecticide resistance in control campaigns. These results highlight a growing trend of pyrethroid resistance in *Ae. aegypti*, driven by the widespread use of these insecticides. Since pyrethroids are the only insecticides approved for domestic and sanitary use in Argentina, the findings of our study have critical implications for evidence-based public health policies aimed at dengue control. The inclusion of alternative insecticidal and other control strategies should be considered to reduce selection pressure on *Ae. aegypti* populations.

## Conclusions

The work presented here is the consequence of a joint effort involving the local BAP government and scientific researchers, members of the RAReP. Our results indicate that the pressure exerted by pyrethroids led to the emergence and expansion of 1016Ikdr + 1534Ckdr mutations in *Ae. aegypti* from BAP. We have also detected V410L mutation in central Argentina for the first time to our knowledge, indicating that, as in other countries of the region, pyrethroid resistance is a serious problem. We also improve the processivity and accuracy of genotyping methods. The results are both a tool for resistance monitoring and a sign of alarm to direct efforts towards finding sustainable methods for vector control to complement or replace pyrethroids shortly. Joint efforts between academia and authorities to develop and implement public policies for vector control are a productive way to transfer scientific results for their application in public health.

## Supplementary Information

Supplementary material 1.

## Data Availability

No datasets were generated or analyzed during the current study.

## Abbreviations

BAP: Buenos Aires Province
HRM: High-resolution melting
HWE: Hardy-Weinberg equilibrium
*Kdr*: Knockdown resistance mutations
mHRM: Multiplex high-resolution melting
MT: Melting temperature
SR: Sanitary region
VGSC: Voltage-gated sodium channel
WHO: World Health Organization
